# Enhancing butanol tolerance of *Escherichia coli* reveals hydrophobic interaction of multi-tasking chaperone SecB

**DOI:** 10.1186/s13068-019-1507-7

**Published:** 2019-06-28

**Authors:** Guochao Xu, Anning Wu, Lin Xiao, Ruizhi Han, Ye Ni

**Affiliations:** The Key Laboratory of Industrial Biotechnology, Ministry of Education, School of Biotechnology, Jiangnan University, Wuxi, 214122 Jiangsu China

**Keywords:** Molecular chaperone engineering, Butanol tolerance, SecB, Random mutagenesis, Hydrophobic interaction

## Abstract

**Background:**

*Escherichia coli* has been proved to be one promising platform chassis for the production of various natural products, such as biofuels. Product toxicity is one of the main bottlenecks for achieving maximum production of biofuels. Host strain engineering is an effective approach to alleviate solvent toxicity issue in fermentation.

**Results:**

Thirty chaperones were overexpressed in *E. coli* JM109, and SecB recombinant strain was identified with the highest *n*-butanol tolerance. The tolerance (*T*) of *E. coli* overexpressing SecB, calculated by growth difference in the presence and absence of solvents, was determined to be 9.13% at 1.2% (v/v) butanol, which was 3.2-fold of the control strain. Random mutagenesis of SecB was implemented and homologously overexpressed in *E. coli*, and mutant SecB_T10A_ was identified from 2800 variants rendering *E. coli* the highest butanol tolerance. Saturation mutagenesis on T10 site revealed that hydrophobic residues were required for high butanol tolerance of *E. coli*. Compared with wild-type (WT) SecB, the *T* of SecB_T10A_ strain was further increased from 9.14 to 14.4% at 1.2% butanol, which was 5.3-fold of control strain. Remarkably, *E. coli* engineered with SecB_T10A_ could tolerate as high as 1.8% butanol (~ 14.58 g/L). The binding affinity of SecB_T10A_ toward model substrate unfolded maltose binding protein (preMBP) was 11.9-fold of that of WT SecB as determined by isothermal titration calorimetry. Residue T10 locates at the entrance of hydrophobic substrate binding groove of SecB, and might play an important role in recognition and binding of cargo proteins.

**Conclusions:**

SecB chaperone was identified by chaperone mining to be effective in enhancing butanol tolerance of *E. coli*. Maximum butanol tolerance of *E. coli* could reach 1.6% and 1.8% butanol by engineering single gene of SecB or SecB_T10A_. Hydrophobic interaction is vital for enhanced binding affinity between SecB and cargo proteins, and therefore improved butanol tolerance.

**Electronic supplementary material:**

The online version of this article (10.1186/s13068-019-1507-7) contains supplementary material, which is available to authorized users.

## Background

Synthetic biology is becoming a promising alternative to synthetic chemistry and has enormous implications for the production of natural products, fine and bulk chemicals, drugs, biofuels, etc. [1, 2]. *Escherichia coli* has proved to be one of the most important platform microorganism for synthetic biology, considering its diverse genetic engineering tools for pathway and module reconstruction, genomic sequence information for metabolic engineering and rational design, and recently developed computational tools for process optimization [3, 4]. Due to the extensive consumption of fossil fuels, renewable biofuels such as biobutanol have become attractive alternatives. *E. coli* has been employed in the synthesis of organic solvent-like chemicals including gasoline, diesel, aviation fuel, etc. [5].

It is widely known that toxicity of bioproducts (e.g., alcohols, aldehydes, ketones and carboxylic acids) to *E. coli* is one of the main bottlenecks for achieving maximum production of biofuels [6]. Under merely 1.0% butanol (v/v), the growth of *E. coli* could be severely inhibited [7]. Penetration and accumulation of organic solvents in cell membrane and cytoplasm might lead to denaturation of functional proteins, oxidative stress and disorganization of cellular structure, resulting in the loss of ions and changes of intracellular pH and membrane fluidity, the leakage of cytoplasm, and eventually cell death [8]. Engineering host strain for enhanced product tolerance is often envisaged as one of the effective approaches to improve biofuel production [9, 10].

Toxicity effects vary across different organic solvents and are assumed to be closely related to their hydrophobicity (log*P*). Organic solvents with log*P* values between 1 and 5 were found to be particularly toxic due to their similar hydrophobicity to membrane and easy penetration into membrane, especially butanol (0.88, log*P*) [11] ranking as one of the most toxic organic solvents. Exposure of *E. coli* to butanol stress has been shown to lead to oxidative stress, acid stress, heat shock and envelope stress [12, 13]. Microbes have evolved a variety of mechanisms to adapt to toxic butanol such as efflux pump, heat shock proteins, membrane modification, cell morphology and general stress response [14]. Inspired by naturally occurred responses of microbes, various genetic, genomic and synthetic strategies have been developed to alleviate the toxicity, including overexpression of fatty acid biosynthesis genes to keep the integrity and fluidity of membrane, efflux pumps genes to strengthen solvents extrusion [15, 16], molecular chaperones to disaggregate the denatured proteins [17], TCA cycle genes to increase energy supply [18], knockout of some negative regulation genes [19], global transcription machinery engineering (gTME) for systematical optimization [20], adaptive evolution using visualizing evolution in real time (VRET) [21], and additionally, exogenous membrane insertion molecules to keep the constant membrane fluidity [22]. Among them, molecular chaperones were first demonstrated to increase the butanol tolerance of solventogenic *Clostridium acetobutylicum* by 85% through overexpression of *groESL* [23]. Furthermore, GroESL-overproducing *Lactobacillus* and *Lactococcus* strains could grow in the presence of 0.5% (v/v) butanol for 5 h, while the viability of parental strain declined after 1 h [24]. This strategy has been extended to *E. coli*, where coexpression of *groESL* and *clpB* led to dramatic improvements in viability with ethanol, butanol and butane-1,2,4-triol [25]. More recently, the heterologous expression of thermophilic chaperones from thermophilic bacteria has been shown to increase ethanol tolerance of *C. acetobutylicum*, *E. coli* and *Zymomonas mobilis* [26, 27].

Although overexpression of a variety of chaperones might enhance microbial organic solvent tolerance, studies using genomic libraries or global strategies have often failed to identify them as overexpression targets [28, 29]. Only GroESL and ClpB from diverse microorganisms have been reported to alleviate the organic solvent sensitivity of *E. coli* [17]. It is of special interest to identify more chaperones with ability in enhancing butanol resistance, which could be further combined with other strategies for improving the organic solvents tolerance of host cells. In this study, systematical characterization of molecular chaperones of *E. coli* was carried out to identify functional chaperones related to butanol stress response, enhance viability of host cells under organic solvent challenges, and further understand the mechanisms of chaperones in butanol tolerance.

## Results

### Mining for molecular chaperones related to butanol tolerance

In our preliminary study, chaperone Spy, an important component of Bae and Cpx response systems, exhibited positive role in enhancing butanol tolerance of *E. coli* JM109 [30]. Additionally, chaperones GroEL and ClpB have been reported to be capable of alleviating butanol sensitivity of *E. coli* [25]. Inspired by above observations, we attempted to identify molecular chaperones for improving the robustness of *E. coli* to high butanol titer in this study. Thirty chaperones responsible for folding/assembly (folding, refolding, disaggregating, isomerize) and localization (cytosolic, periplasmic or membrane anchored) were selected from *E. coli* to evaluate their roles in butanol tolerance. These chaperones were NlpE, HybE, RavA, YcaL, ClpA, ClpX, CbpA, HscC, HslO, IbpA, IbpB, NfuA, PpiD, Skp, SecB, SurA, YcdY, YegD, YrhB, ClpB, HchA, GrpE, HtpG, GroEL, LolA, DjlA, BepA, YajL, DnaK and DnaJ. Although some chaperones were dependent on the co-chaperones (such as DnaK with DnaJ), solo overexpression was implemented to discern the roles of each chaperones, since the co-chaperones exist in host cells. Potential function and accession number of these chaperones are summarized in Additional file 1: Table S1. The genes coding for above chaperones were cloned and overexpressed in *E. coli* JM109. SDS-PAGE analysis showed that apparent bands were migrated at the appropriate position, indicating that all the chaperones have been successfully expressed (Additional file 1: Fig. S1). The recombinant *E. coli* strains were further evaluated for butanol tolerance.

**Fig. 1:**
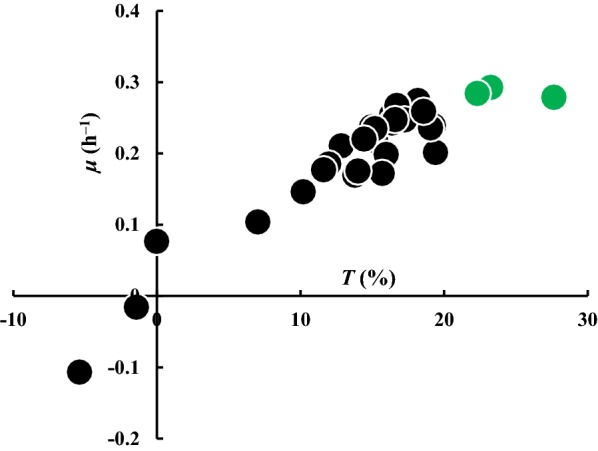
Screening result of *E. coli* strains overexpressed with chaperones identified by genome mining. *μ* denotes specific growth rate between 0 and 2 h (Eq. 1), *T* denotes tolerance to butanol (Eq. 2). All experiments were performed in triplicate. Each dot represents one engineered strain. Green dots: SecB, ClpB and YcdY. Growth curves of all the engineered strains are available in the Additional file

Butanol tolerance assay was performed under 0.8% (v/v) butanol, where specific growth rate (*μ*) between 0 and 2 h (Eq. 1) and the tolerance to butanol (*T*) at 10 h were calculated (Eq. 2). The *μ* indicates the growth rate at initial 2 h, while the *T* represents the maximum growth ability under different butanol concentrations. The growth of *E. coli* JM109/pQE80L (empty) and engineered *E. coli* JM109 without butanol stress was regarded as control, and the screening results are shown in Fig. 1 and Additional file 1: Fig. S2. Under 0.8% butanol, the *μ* and *T* of the control (*E. coli* JM109/pQE80L) were 0.153 h^−1^ and 16.2%. Among all 30 chaperones tested, 19 recombinant strains displayed increased butanol tolerance, while 11 exhibited decreased tolerance. Apparently, overexpression of DjlA, LolA and PpiD severely inhibited the cell growth, with *μ* and *T* lower than 0, especially for PpiD (peptidyl–prolyl *cis*–*trans* isomerase), giving *μ* of − 0.07 h^−1^ and *T* of − 5.38%. Chaperones SecB, ClpB and YcdY, achieved the highest butanol tolerance of *E. coli*, which are protein export chaperone, protein disaggregation chaperone, and putative chaperone, respectively. The *μ* and *T* of SecB, ClpB and YcdY were 0.249 h^−1^ and 27.5%, 0.196 h^−1^ and 24.0%, 0.220 h^−1^ and 21.5%. Remarkably, *E. coli* harboring SecB displayed the highest butanol tolerance; the values of *μ* and *T* were 1.6- and 1.7-fold of those of control strain.

Further evaluation on SecB, ClpB and YcdY was carried out at 1.0% and 1.2% butanol (Table 1 and Additional file 1: Fig. S3). For SecB, the *μ* and *T* at 1.0% and 1.2% butanol were 0.198 h^−1^ and 20.0%, 0.136 h^−1^ and 9.13%, which were 1.7- and 2.0-fold, and 7.2- and 3.2-fold of those of the control. ClpB has been reported to be effective in enhancing the butanol tolerance of *E. coli* by 25% growth rate increase at 1.0% butanol [31]. Herein, the *μ* of ClpB strain was 0.139 h^−1^ at 1.0% butanol, 16% higher than 0.120 h^−1^ of the control strain. To the best of our knowledge, the positive effect of chaperones SecB and YcdY in the microbial tolerance toward butanol was discovered for the first time. SecB is a component of well-known Sec-dependent pathway, and the interaction between SecB and SecA is vital for protein translocation. Therefore, coexpression of SecA and SecB was firstly constructed in *E. coli*. According to the quantitative PCR result, mRNA levels of *secA* and *secB* under 0.8% and 1.2% butanol were 13.8- and 7.48-fold, and 9.56- and 6.68-fold higher than those of *E. coli* JM109 as calculated by 2^−ΔΔ*C*t^ method, suggesting the successful coexpression of SecA and SecB. However, no improvement in host butanol tolerance was observed for SecA–SecB-coexpressed strain (Additional file 1: Fig. S4), suggesting that the role of SecB in butanol tolerance might be SecA independent, since SecB is multitasking [32]. Nevertheless, considering its outstanding promoting effect, SecB was selected for protein engineering to understand its functional patterns and further enhance the butanol tolerance of host cells.

**Table 1:** Growth kinetic parameters of engineered *E. coli* toward different concentrations of butanol

Strain	0.8% Butanol		1.0% Butanol		1.2% Butanol	
	*μ*^a^/h^−1^	*T*^b^/%	*μ*/h^−1^	*T*/%	*μ*/h^−1^	*T*/%
Control	0.153 ± 0.009	16.2 ± 0.2	0.120 ± 0.007	10.1 ± 0.6	0.019 ± 0.009	2.86 ± 0.40
SecB	0.249 ± 0.027	27.5 ± 0.2	0.198 ± 0.018	20.0 ± 0.8	0.136 ± 0.016	9.13 ± 0.21
ClpB	0.196 ± 0.021	24.0 ± 1.2	0.139 ± 0.012	15.5 ± 1.2	0.074 ± 0.006	6.65 ± 0.22
YcdY	0.220 ± 0.014	21.5 ± 2.4	0.169 ± 0.021	12.3 ± 0.6	0.062 ± 0.005	6.53 ± 0.21

^a^*μ* denotes specific growth rate between 0 and 2 h (Eq. 1)

^b^*T* denotes tolerance to butanol (Eq. 2); All experiments were performed in triplicate. Growth curves of all the tested strains are available in Additional file

### Random mutagenesis of secB for higher butanol tolerance

**Fig. 2:**
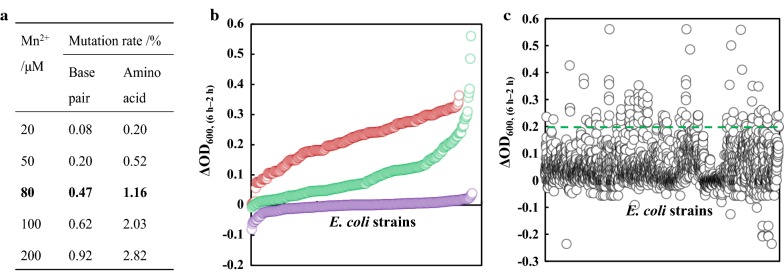
Development of SecB random mutagenesis library and high-throughput screening. **a** Effect of Mn^2+^ concentrations on mutation rate of SecB. **b** Effect of butanol concentration on high-throughput screening. **c** High-throughput screening of SecB library containing 2800 variants. Red dot: 0.8% butanol stress, green dot: 1.0% butanol stress, purple dot: 1.2% butanol stress. Green dotted line: value of the WT SecB strain

From the crystal structure of SecB (PDB: 1QYN), holoenzyme of SecB is a homotetramer that assembles as a dimer of dimers [33, 34]. The substrates of SecB, generally known as preproteins, is usually recognized and exported through the 70-Å-long hydrophobic channels [35]. Error-prone PCR was performed for the directed evolution of SecB. By varying the concentrations of Mn^2+^ from 20 μM to 200 μM, the mutation rates in both base pair and amino acid increased accordingly (Fig. 2a). Obviously, Mn^2+^ of 80 μM was suitable for the evolution of SecB, with mutation rate of 0.47% (base pair) and 1.16% (amino acid).

To achieve high-throughput screening of the random mutagenesis library of SecB, a suitable butanol concentration is important, because higher butanol stress leads to cell death, while lower butanol stress leads to difficulty in distinguishing growth rate. About 900 colonies were selected for optimization of butanol concentrations ranging from 0.8 to 1.2%. As illustrated in Fig. 2b, 1.2% butanol is too severer for the growth of *E. coli* and most of *E. coli* stopped growing, while 0.8% butanol is more moderate since the growth rates of all the tested variants were similar. In 1.0% butanol, the difference in growth was evident, with ΔOD_600_ from 0 to 0.5. Consequently, 1.0% butanol was regarded as the appropriate butanol stress for the high-throughput screening of SecB random mutagenesis library. Furthermore, about 2800 *E. coli* strains harboring SecB mutants were picked up from the random mutagenesis library for screening. Only 48 mutants displayed significant increase in butanol tolerance as summarized in Additional file 1: Table S5. Secondary screening of above 48 strains was carried out in shaking flasks in triplicate. Two mutants, strains 7C4 and 26E3, displayed the highest tolerance in 1.0% butanol. According to the sequencing result, mutations in 7C4 and 26E3 were T10A and E8D, respectively, and these two mutants were designated as SecB_T10A_ and SecB_E8D_. When further increased the butanol concentration to 1.2%, SecB_T10A_ strain displayed 138% and 57% increase in *μ* and *T*, while the growth of SecB_E8D_ strain was similar to that of wild-type SecB. As a result, SecB_T10A_ was proved to be effective in enhancing robustness of *E. coli* under butanol stress. To verify whether the T10A mutation led to the higher expression level of SecB and therefore the increase of *μ*, quantitative PCR was implemented (Additional file 1: Fig. S5). The result suggests that T10A mutation had little influence on the expression level of SecB according to the fold changes of mRNA.

### Saturation mutagenesis on T10 of secB

**Fig. 3:**
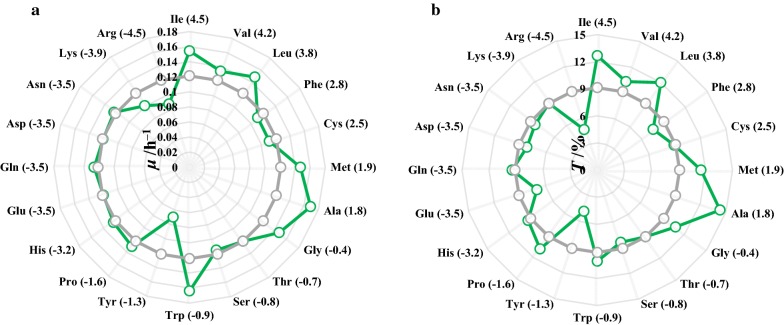
Effect of saturation mutagenesis at T10 of SecB on *E. coli* JM109 under 1.2% butanol. **a** Specific growth rate (*μ*). **b** Tolerance to butanol (*T*). Values of *μ* and *T* were depicted as hollow dots in the radar map, green dot: *μ* and *T* of SecB variants, gray dot: *μ* and *T* of WT SecB, numbers in the bracket were hydrophobic index of the twenty amino acids. Detailed growth curves of all the mutation strains are available in Additional file

To better understand the role of T10, saturation mutagenesis was performed at T10 residue of SecB by whole-plasmid PCR. The *E. coli* transformants containing SecB variants were evaluated toward 1.2% butanol (Additional file 1: Fig. S6). It was illustrated that mutation of threonine into hydrophobic amino acids favored the higher butanol tolerance of *E. coli*, such as isoleucine (hydrophobicity index of 4.5), valine (4.2), leucine (3.8), methionine (1.9) and alanine (1.8), while mutation into hydrophilic tyrosine (− 1.3) and arginine (− 4.5) led to the significantly decreased growth rate and butanol tolerance (Fig. 3). Among them, the T10A mutant ranked the highest growth rate and butanol tolerance, with *μ* of 0.169 h^−1^and *T* of 14.3%. For other hydrophobic mutants, slightly lower *μ* and *T* were obtained, specifically, T10I (0.154 h^−1^ and 12.7%), T10L (0.148 h^−1^ and 12.0%), T10M (0.147 h^−1^ and 11.5%). Whereas for hydrophilic mutants T10Y and T10R, the lowest *μ* (0.070 h^−1^ and 0.088 h^−1^) and *T* (4.8% and 4.7%) were noted. Consequently, a hydrophobic pattern was obviously vital for butanol tolerance according to the saturation mutagenesis of T10, except for phenylalanine, a sterically hindered hydrophobic amino acid.

**Fig. 4:**
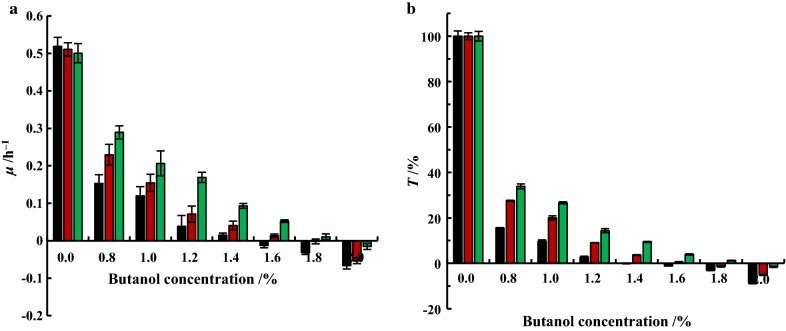
Maximum butanol tolerance evaluation of *E. coli* harboring SecB and SecB_T10A_. **a** Specific growth rate (*μ*). **b** Tolerance to butanol (*T*). Black bar: *E. coli* JM109/pQE80L; red bar: *E. coli* JM109/pQE80L–*secB*; green bar : *E. coli* JM109/pQE80L–*secB*_*T10A*_. All experiments were carried out in triplicate

Maximum butanol tolerance of *E. coli* harboring SecB and SecB_T10A_ was also tested against 0.8−2.0% butanol. Without butanol, *E. coli* strains harboring pQE80L (control), pQE80L–*secB* and pQE80L–*secB*_*T10A*_ displayed similar growth status and OD_600_ could reach 4.94, 4.72 and 4.71. As shown in Fig. 4 and Additional file 1: Fig. S7, along with the increase of butanol, both the growth and tolerance rates decreased. For the control strain, OD_600_ did not increase at 1.4% butanol with tolerance (*T*) of 0 within 10 h. The *μ* and *T* slightly decreased at 1.6% butanol to lower than 0. All above indicated that the maximum butanol-tolerant concentration of *E. coli* JM109 was 1.4%. SecB strain was able to grow at 1.6% butanol with *μ* and *T* of 0.013 h^−1^ and 0.53%, while no cell growth at 1.8% butanol and with *μ* and *T* below than 0. As expected, SecB_T10A_ strain exhibited the highest tolerance among 3 strains, a slightly increased OD_600_ was observed at 1.8% butanol with *μ* and *T* of 0.012 h^−1^ and 1.21%. At 2.0% butanol, the *μ* and *T* of SecB_T10A_ strain were below than 0, while the *μ* of SecB and control strains was − 0.078 h^−1^ and − 0.035 h^−1^, respectively. Consequently, SecB and SecB_T10A_ could enhance the butanol tolerance of *E. coli* from 1.4% (control) to 1.6−1.8%.

### Tolerance assay of SecB and SecB_T10A_ toward diverse solvents

To further expand the application of SecB_T10A_, the tolerance against diverse organic solvents with log*P* values ranging from − 1.0 to 3.2 were determined (Table 2). Solvents, *i*-butanol (log*P *= 0.76), toluene (2.5), and cyclohexane (3.2), were unfavorable for the cell growth, while DMF (− 1.0), acetonitrile (− 0.33), ethanol (− 0.24) and *i*-propanol (0.39) exhibited better biocompatibility. In the presence of 0.8% octanol, all three strains could not grow at all (Additional file 1: Fig. S8). Except for acetone and cyclohexane, SecB_T10A_ strain showed higher tolerance toward all challenged solvents compared with SecB strain. At 1.2% butanol, the *μ* and *T* of SecB_T10A_ were 0.169 h^−1^ and 14.4% which were 1.2- and 1.6-fold, 8.9- and 5.3-fold of the SecB and control strains, respectively. Compared with *n*-butanol, toxicity of *i*-butanol was much lower, resulting in higher growth rates and tolerance of all three strains, even under 3.0% *i*-butanol (Table 2). In summary, SecB and SecB_T10A_ displayed higher *μ* and *T* to all the tested solvents, especially to some short-chain alcohols.

**Table 2:** Tolerance of *E. coli* harboring SecB and SecB_T10A_ toward diverse solvents

Solvent^a^	log*P*	*E. coli* JM109/pQE80L			*E. coli* JM109/pQE80L-*secB*			*E. coli* JM109/pQE80L-*secB*_*T10A*_		
		*μ*^b^/h^−1^	*T*^c^/%	*A*^d^/%	*μ*/h^−1^	*T*/%	*A*/%	*μ*/h^−1^	*T*/%	*A*/%
PBS	–	–	–	14 ± 1	–	–	15 ± 1	–	–	16 ± 1
DMF	− 1.0	0.294 ± 0.015	42.4 ± 1.9	17 ± 1	0.462 ± 0.015	56.6 ± 1.1	17 ± 5	0.520 ± 0.014	65.0 ± 1.7	17 ± 3
Acetonitrile	− 0.33	0.372 ± 0.014	35.4 ± 0.9	18 ± 2	0.511 ± 0.022	76.3 ± 1.7	18 ± 1	0.430 ± 0.012	76.9 ± 2.5	18 ± 2
Ethanol	− 0.24	0.418 ± 0.011	51.1 ± 0.3	21 ± 8	0.420 ± 0.012	60.6 ± 1.0	23 ± 2	0.408 ± 0.013	64.3 ± 0.4	23 ± 1
Acetone	− 0.23	0.100 ± 0.004	11.9 ± 0.4	16 ± 1	0.183 ± 0.013	14.3 ± 0.1	18 ± 6	0.114 ± 0.009	12.6 ± 0.7	18 ± 11
*i*-Propanol	0.39	0.121 ± 0.006	14.2 ± 0.4	23 ± 1	0.244 ± 0.012	20.0 ± 0.2	24 ± 3	0.251 ± 0.012	24.9 ± 0.2	26 ± 7
*i*-Butanol	0.76	0.083 ± 0.008	7.27 ± 0.28	13 ± 1	0.223 ± 0.011	13.1 ± 0.1	13 ± 5	0.235 ± 0.012	14.7 ± 0.2	12 ± 1
*n*-Butanol	0.88	0.019 ± 0.005	2.86 ± 0.40	14 ± 1	0.136 ± 0.016	9.13 ± 0.21	13 ± 1	0.169 ± 0.013	14.4 ± 0.9	11 ± 1
Toluene	2.5	0.016 ± 0.004	1.19 ± 0.18	1.9 ± 0.6	0.046 ± 0.008	4.83 ± 0.21	1.4 ± 0.8	0.071 ± 0.006	6.12 ± 0.21	1.3 ± 0.9
Cyclohexane	3.2	0.026 ± 0.006	0.59 ± 0.21	4.8 ± 1.1	0.067 ± 0.007	3.11 ± 0.23	3.5 ± 0.8	0.038 ± 0.005	2.27 ± 0.29	2.6 ± 1.9

^a^Solvent concentration: 3% *N*,*N*-dimethylformamide (DMF), 3% acetonitrile, 3% ethanol, 1% acetone, 3% *i*-propanol, 3% *i*-butanol, 1.2% *n*-butanol, 0.2% toluene and 0.8% cyclohexane

^b^*μ* denotes specific growth rate between 0 and 2 h (Eq. 1)

^c^*T* denotes tolerance to butanol (Eq. 2)

^d^*A* denotes adhesion rate of solvents (Eq. 3). All experiments were performed in triplicate. Growth curves of all the tested strains are available in Additional file

The surface hydrophobicity of the engineered *E. coli* was also determined by microbial adhesion to solvents (MATS) method [36]. Adhesion (*A*) of engineered *E. coli* toward different solvents could be used to estimate the surface hydrophobicity. For hydrophobic solvents, higher *A* represents a higher similarity in hydrophobicity of cell surface and solvents, resulting in higher absorbance of solvents and higher inhibition on cell growth, and vice versa. As shown in Table 2, the *A* of *E. coli* harboring SecB and SecB_T10A_ was 13% and 11% under butanol stress, lower than that of the control (14%), which also explains their improved butanol tolerance. However, for the hydrophilic solvents, such as ethanol, acetone and *i*-propanol, the *A* of SecB and SecB_T10A_ strains was higher than those of *E. coli* JM109. Also, higher *A* was observed toward solvents with lower log*P* value. Our results illustrated that the cell surface of SecB and SecB_T10A_ strains was more hydrophilic than the *E. coli* control, which is conducive to less absorption of hydrophobic solvents.

### ITC assay of SecB and SecB_T10A_ toward preMBP

**Fig. 5:**
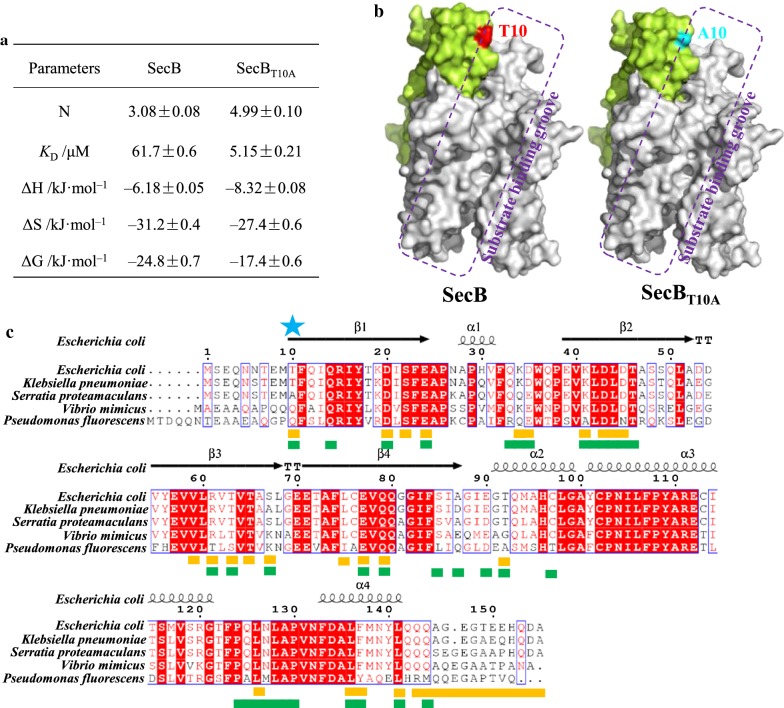
ITC result and consensus analysis SecB and homology proteins. **a** ITC result between SecB or SecBT10A and preMBP. N represents stoichiometric number. **b** Comparison of T10 and A10 in homotetramer of SecB. One subunit was depicted in green color, T10 and A10 were drawn in red and blue color, respectively, and substrate binding groove was illustrated with purple dotted square. **c** Multiple sequence alignment between SecB and homology proteins. Blue star: T10 site, green bar: residues interacted with SecA, yellow bar: residues involved in interaction with substrate. SecB homology proteins are from *Escherichia coli* (Accession No.: P0AG86), *Klebsiella pneumoniae* (A6TFK4), *Serratia proteamaculans* (A8GLB9), *Vibrio mimicus* (D2YLC5) *Pseudomonas fluorescens* (WP_060765829.1)

Since the main function of SecB is protein export, SecB and SecB_T10A_ might replenish more proteins to replace the denatured proteins caused by butanol stress. Isothermal titration calorimetry (ITC) experiment was performed to measure the binding affinity between SecB and substrate proteins. The unfolded precursor form of maltose binding protein (preMBP) is one model substrate for SecB, and was selected for ITC experiment. preMBP was prepared by two rounds of PCR, in which the pre-signal was fused to MBP. SecB, SecB_T10A_ and preMBP were purified through nickel affinity chromatography. According to the SDS-PAGE in Additional file 1: Fig. S9, all three proteins were purified to homogeneity. The suitable ratio of SecB or SecB_T10A_ to preMBP was optimized to be 6:1. ITC was carried out by titration of SecB or SecB_T10A_ into preMBP at 25 °C. As shown in Fig. 5a and Additional file 1: Fig. S10, the stoichiometric number (N) of SecB_T10A_ toward preMBP increased to 4.99 ± 0.15 from 3.08 ± 0.11 of WT SecB, and the equilibrium dissociation constant (*K*_D_) of SecB_T10A_ significantly decreased to 5.2 ± 0.3 μM from 61.7 ± 1.8 μM of WT SecB, indicating 11.9-fold increase of binding affinity toward preMBP. It was apparent that SecB_T10A_ displayed increased heat changes (ΔH) than WT SecB from − 6.18 ± 0.31 to –8.32 ± 0.67 kJ mol^−1^ during the reaction, however, a considerable decrease in entropy (ΔS) from − 31.2 ± 2.5 to − 27.4 ± 1.6 kJ mol^−1^ as shown in Fig. 5a. The Gibbs free energy difference (ΔG) of SecB_T10A_ was − 17.4 ± 0.9 kJ mol^−1^, while the ΔG of WT SecB was − 24.8 ± 1.5 kJ mol^−1^. All above proved that the specific binding of SecB to preMBP was enthalpy-driven and the mutation of threonine at residue 10 into alanine increased the binding affinity toward cargo protein.

From the crystal structure (Fig. 5b) and sequence alignment (Fig. 5c) of SecB, T10 is located at the entrance of substrate binding groove, which binds to the substrate protein by hydrophobic interaction. T10 is not a conserved residue, and has been reported to participate in the interaction between preproteins and SecA [32]. In SecB from *Serratia proteamaculans* (A8GLB9), same alanine appears at residue 10. While in SecB from *Vibrio mimicus* (D2YLC5) and *Pseudomonas fluorescens* (WP_060765829.1), it turns out to be glutamine.

## Discussion

Engineering of host tolerance is important for synthetic biology, especially in the synthesis of chemicals employing *E. coli*, such as butanol [9]. Butanol is one of the promising biofuels with advantages such as higher energy density, lower volatility and less corrosion [4]. However, butanol is highly toxic to most microorganisms. A variety of strategies have been adopted in engineering the butanol tolerance of *E. coli* (Table 3), which are possible solutions for improving butanol titer [7]. Although, it is widely accepted that the solvent tolerance of host cells is beyond solo gene [37], the study of individual gene involved in solvent tolerance is also necessary to unveil the complicated mechanisms. Solo genes such as *groESL*, *OmpC*-*TMT*, and *acrB*_*I466F/M355L/S880P*_ have been overexpressed in *E. coli* to achieve tolerance against 0.7–1.5% butanol, and these genes participate in prompting protein folding, scavenging of intracellular and extracellular free radicals and enhancing efflux pump (Table 3).

**Table 3:** Engineering strategies to improve butanol tolerance of *E. coli*

Entry	Strategy	Maximum butanol concentration (v/v)	Function or mechanism	References
1	Overexpression of *CRP* variants	1.2% butanol	Global transcription factor, 308 genes with > 2.0-fold difference in expression level	[38, 39]
2	Overexpression of *groESL*	1.0% butanol	Folding both nascent and misfolded proteins	[25]
3	Overexpression of *OmpC*-*TMT*	1.5% butanol	Scavenging of intracellular and extracellular free radicals	[40]
4	Knockout of *lon* and *proV*	2.0% butanol	High expression of AcrAB–TolC pump	[19]
5	Overexpression of *acrB*_*I466F/M355L/S880P*_	25% increase at 0.7% butanol	Promoting secretion of butanol from the cell	[15]
6	Overexpression of *ATF, fabD, feoA* and *srpABC*	2.0% butanol	Synergistic effect of artificial transcription factor, fatty acid synthesis, iron-uptaking protein FeoA, efflux pump SrpABC from *Pseudomonas putida*	[41]
7	Overexpression of *rpoD*	2.0% butanol	Global transcription factor, 197 upregulated genes and 132 downregulated genes	[20]
8	Overexpression of *secB*_*T10A*_	1.8% butanol (5.3-fold improvement in *T* at 1.2% butanol)	Exporting nascent unfolded protein, improved binding affinity of substrate proteins	This study

Molecular chaperones could be classified into foldase (e.g., DnaK and groEL), holdase (e.g., IbpB and SecB), and disaggregase (e.g., ClpB). Misfolding or aggregation of proteins, resulted by premature termination, miss-folding or stress, is assisted by chaperones to favor proper folding, refolding partially fold proteins, dissolve aggregates, dispose and replenish of irretrievably damaged proteins [42]. However, only GroEL and ClpB from *E. coli* [7], GroESL from *C. acetobutylicum* [43], *Cupriavidus necator* [44] and *Lactococcus lactis* [24], and PhaP from *Azotobacter* sp. strain FA8 with chaperone-like properties [10] have been proved to be effective in enhancing the host tolerance against butanol. As a result, genome mining for more molecular chaperones in improving the butanol tolerance of *E. coli* was of special interest. SecB was the most effective even at higher butanol concentration (1.2%) after screening. SecB is a component of Sec-dependent pathway; general proteins secretion system consisted of SecA, SecB and SecYEG [45]. Although generic chaperones DnaJ–DnaK (Hsp40–Hsp70), GroES–GroEL (Hsp10–Hsp60) and trigger factors can export proteins in *E. coli*, a vast majority of proteins are translocated across the cytoplasmic membrane by this Sec-dependent pathway. SecB is a highly acidic proteins, and has a long substrate binding groove, which is highly hydrophobic and recognizes the nine amino acid motif enriched in aromatic and basic residues [46]. This special structure of SecB dedicates to export preproteins with specific properties. Based on the pulse labeling protein secretion studies and comparative proteomics, a number of preproteins of SecB have been identified, including MBP, OmpA, OmpF, OmpT, OmpX, TolB, TolC, PhoE, LamB, etc., some of which are important for the resistance against solvent stress such as TolB and TolC [15]. Besides protein exportation and translocation, SecB also plays important role in anti-folding [47] and stress-responsive toxin–antitoxin system [46], indicating it is multitasking. Herein, the role of SecB in endowing *E. coli* with high tolerance against organic solvents was discerned.

Overexpression of SecB in *E. coli* could improve the butanol tolerance. Under 1.2% butanol, the specific growth rate (*μ*) and tolerance rate to butanol (*T*) were 3.6- and 3.2-fold of *E. coli* JM109. The maximum butanol tolerance increased from to 1.6%. Furthermore, random mutagenesis was performed on SecB, and one mutant SecB_T10A_ was obtained with enhanced effect on butanol tolerance. Saturation mutagenesis at T10 revealed that most hydrophobic amino acids displayed similar effect, while hydrophilic amino acids led to decreased specific growth rate and tolerance rate. This T10 site locates at the entrance of hydrophobic substrate binding groove and is not conserved in SecB homologous proteins from different microorganism. The mutation of threonine into alanine was supposed to enhance the hydrophobicity of substrate binding groove and further improve the binding affinity and export efficiency toward cargo proteins. ITC experiment revealed that about 11.9-fold increase in binding affinity (*K*_D_) was found in interaction between SecB_T10A_ and preMBP, which was consistent with the result of L42R mutation of SecB [48]. The increased Δ*H* and decreased Δ*S* of SecB_T10A_ strain suggested that T10A mutation reduced the mobility of SecB in preMBP binding and increased the proportion of energy supply during the reaction. In the case of mutation threonine into the hydrophobicity phenylalanine, the bulk phenylalanine at the entrance of substrate binding groove might interfere the binding process, which should be responsible for the decreased butanol tolerance of SecB_T10F_ strain. Also, this unconserved T10 is an effective position for the engineering of SecB homology proteins for the secretion of cargo proteins. Finally, the butanol tolerance of *E. coli* could be further increased to as high as 1.8% butanol by solo SecB_T10A_. This engineered *E. coli* strain harboring SecB_T10A_ displayed higher tolerance against *i*-butanol, *n*-butanol, and toluene. The surface of engineered *E. coli* cells is more hydrophilic than that of the *E. coli* control, which is unfavorable for the absorption of hydrophobic solvents and their penetration across the cell membrane. Recently, chaperone SecB was also evolved to improve the ability in controlling the toxin–antitoxin system of *E. coli* by directed evolution. Substitution of residues located in substrate binding tunnel was found to be important in modulation the binding of specific substrates without affecting the binding of presecretory proteins [35]. All above proved that this SecB is multi-tasking and the hydrophobic interaction was very important for the translocase function of SecB and enhancing the butanol tolerance of *E. coli*.

## Conclusions

Thirty chaperones were evaluated for their functions in butanol tolerance of *E. coli*, and SecB chaperone was proved to be the most efficient, with the highest specific growth rate (*μ*) and tolerance (*T*) even at 1.2% (v/v) butanol. Random mutagenesis of SecB revealed that T10A mutation was important for enhancing the binding affinity toward substrate proteins and further improving the butanol tolerance of *E. coli*. Saturation mutagenesis on T10 indicates a hydrophobic function of this site. With SecB_T10A_, recombinant *E. coli* could tolerate as high as 1.8% butanol. In the future, this SecB_T10A_ could be combined with other butanol tolerance prompting components and more SecB homology proteins from different microorganism could be mined for further engineering the organic solvent tolerance of *E. coli*. Moreover, the potential of the SecB engineered *E. coli* would be evaluated and optimized in biobutanol synthesis by introducing clostridia coenzyme A-dependent butanol producing pathway constructed in our previous study [49].

## Methods

### Chemical reagents, bacterial strain, plasmids and medium

*n***-**Butanol, methanol, ethanol, *i*-propanol, *i*-butanol, toluene, cyclohexane, acetone, tetrahydrofuran, acetonitrile and dimethylformamide (DMF) were analytical grade and purchased from Sigma Aldrich. Plasmid pQE80L carrying T5 promoter and kanamycin-resistant gene was bought from Novagen and utilized for chaperones overexpression.

### General protocol for clone and overexpression of chaperones

Molecular chaperones were overexpressed to evaluate their role in resistance against organic solvents. Thirty molecular chaperones coding genes (*nlpE*, *hybE, ravA, ycaL*, *clpA*, *clpX*, *cbpA*, *hscC*, *hslO*, *ibpA*, *ibpB*, *nfuA*, *ppiD*, *skp*, *secB*, *surA*, *ycdY*, *yegD*, *yrhB*, *clpB*, *hchA*, *grpE*, *htpG*, *groL*, *lolA*, *djlA*, *bepA*, *yajL*, *dnaK* and *dnaJ*) were selected and cloned with genomic DNA of *E. coli* JM109 and primers as listed in Additional file 1: Table S1. All the PCR products were ligated into pQE80L digested with *Bam*HI and *Hin*dIII (Takara Ltd Ltd, Shanghai) using Exanse II (Vazyme Ltd, Nanjing). The recombinant plasmids were transformed into *E. coli* JM109 to form the engineered strains. All the chaperones were verified by digestion with *Bam*HI and sequencing.

Each colony of engineered *E. coli* JM109 was picked up and inoculated into LB medium and cultivated at 37 °C and 180 rpm overnight. Then, 1% (v/v) culture was transferred into 30-mL fresh LB medium (10 g L^−1^ tryptone, 5 g L^−1^ yeast extract, 10 g L^−1^ NaCl, pH 7.0) and further cultivated at 37 °C and 180 rpm. When the OD_600_ reached to 0.3, 0.2-mM isopropyl *β*-d-1-thiogalactopyranoside (IPTG, Takara) was added and incubated at 30 °C and 180 rpm for 6 h. Then, the cells were collected via centrifugation at 8800×*g* and 4 °C, and disrupted using high-pressure homogenizer (ATS BASIC-II, Shanghai). Chaperones were verified by SDS-PAGE.

### General protocol for solvent tolerance assay

Tolerance to organic solvents was determined according to the growth status of each strain. When the OD_600_ reached to 0.3, 0.2-mM IPTG was added to induce the chaperones expression. Furthermore, when the OD_600_ reached to 0.7–0.8, 1.0% (v/v) n-butanol or other solvents was added into the culture. Samples were withdrawn at 2-h interval and growth status of strains was monitored at 600 nm.

### Calculation of growth kinetic parameters and statistical analysis

Specific growth rate (*μ*) of *E. coli* strain was calculated according to Eq. 1 [10]. Tolerance (*T*) to solvents was determined based on the growth of engineered *E. coli* in the presence and absence of solvent as shown in Eq. 2. All the experiments were independently performed for at least three times and the average values and standard deviation are exhibited. Significant difference analysis was evaluated by *t* test with a *P* value < 0.05 as significant difference.

1 $$\mu \,({{\text{h}}^{ - 1}}) = \frac{{\ln {\text{OD}}_{600(2\,{\text{h}})} - \ln {\text{OD}}_{600(0\,{\text{h}})}}}{(2 - 0)\,{\text{h}}}$$

2 $$\text{Tolerance}\,(T, \% ) = \frac{{\text{OD}_{600(\text{presence of solvent},\,1 0{\text{h}})} - \text{OD}_{600(\text{presence\,of\,solvent},\,0 \text{h})}}}{\text{OD}_{600(\text{absence\,of\,solvent},\,10 \text{h})} - \text{OD}_{600(\text{absence of solvent},\,0 \text{h})}} \times 100\%$$

### Construction of random mutagenesis library of SecB

Directed evolution of SecB was implemented to further improve its performance in organic solvent tolerance. Random mutagenesis library of SecB was developed using error-prone PCR with secB-F and secB-R as primers and pET28–*secB* as template. PCR mixture consisted with rTaq polymerase (Takara Ltd, Shanghai), rTaq polymerase buffer, dNTP, 2 mM MgCl_2_ and 80 μM MnCl_2_ for amino acids mutation rate of 1.16%. PCR procedure was set as: pre-denaturation at 95 °C for 5 min, 30 cycles of 98 °C for 10 s, 55 °C for 30 s and 72 °C for 1 min, and further at 72 °C for 10 min. The resultant PCR products was recovered and digested with *Bam*HI and *Hin*dШ, and inserted into pQE80L using Exnase II. The recombinant plasmids were transformed into *E. coli* JM109 and plated on the LB agar plate. Each mono colony was picked up and inoculated into 96-deep-well plate of LB medium and cultivated at 37 °C and 180 rpm overnight to form the random mutagenesis library of SecB.

### High-throughput screening of SecB random mutagenesis library

SecB library was transferred into fresh 96-deep-well plate with LB medium and cultivated at 37 °C and 180 rpm for 0.5 h; then 0.2 mM IPTG was added and further cultivated for 1.5 h. Furthermore, 0.8%, 1.0% or 1.2% butanol was supplemented into culture and the OD_600_ was monitored at 2-h interval for 12 h. The difference of OD_600_ at 12 h and 6 h was used for high-throughput butanol tolerance screening.

### Site-directed saturation mutagenesis at T10 of SecB

To elucidate the role of SecB in butanol tolerance, saturation mutagenesis at T10 of SecB chaperone was performed using whole-plasmid PCR with primers listed in Additional file 1: Table S3. The resultant PCR products were digested with *Dpn*I to remove parental plasmids and then transformed into *E. coli* JM109. Then, all the *E. coli* JM109 strains containing SecB variants were cultivated as above mentioned and were applied for butanol tolerance evaluation.

### Quantitative PCR of secB and secB_T10A_

Total RNA for quantitative PCR (qPCR) was isolated from cultures with and without pretreatment of 1% butanol using the Simply P Total RNA Extraction Kit (BioFlux, Japan). Reverse transcription PCR step was carried out using Rayscript cDNA Synthesis Kit (Generay Ltd, China). Primers for the qPCR are shown in Additional file 1: Table S2. The cDNA product was diluted into 50-fold. qPCR was performed on the LightCycler^®^480 System using 2 µL of diluted cDNA, SYBR^®^ Premix Ex Taq II (Takara Ltd, Shanghai) and 0.4-µM primers. 16S rRNA gene was used as the housekeeping gene in qPCR. qPCR procedure was set as: 95 °C for 30 s, 40 cycles of 95 °C for 5 s and 55 °C for 30 s. 2^−ΔΔ*C*t^ method was adopted to evaluate the fold changes in gene expression level.

### Determination of adhesion rate using microbial adhesion to solvents method

Microbial adhesion to solvents (MATS) method was employed to determine the adhesion (*A*), which was a parameter to reflect the surface hydrophobicity of cells. Recombinant *E. coli* strains harboring pQE80L, pQE80L–*secB* and pQE80L–*secB*_*T10A*_ were induced with 0.2-mM IPTG for 6 h and the cells were harvested at 8800×*g* and 4 °C. Furthermore, the cell pellets were washed with normal saline and the residual normal saline was thoroughly withdrawn. Then, the cells pellets were re-suspended with PBS buffer (pH 6.0, 100 mM) and diluted to OD_400_ of 0.8–0.9, designated as Abs_0_. 4.8-mL above-mentioned liquids was intensively mixed with 0.8-mL diverse solvents, and kept standing for 15 min at room temperature for phase separation. The absorbance at 400 nm of water phase was determined as Abs_1_. *A* was calculated according to Eq. 3. Control experiment was performed by mixing with 0.8-mL PBS buffer (pH 6.0, 100 mM). All experiments were carried out in triplicate.

3 $${\text{Adhesion }}(A,\,\% ) =\frac{{{\text{Ab}}{{\text{s}}_0} - {\text{Ab}}{{\text{s}}_1}}}{{{\text{Ab}}{{\text{s}}_0}}} \times 100\%$$

### Construction of preMBP and protein purification

It was reported that preMBP was the model substrate protein of SecB, which was unfolded maltose binding protein (MBP) in fusion with a pre-signal peptide containing 19 residues at *N*-terminal. preMBP coding gene was cloned from genomic DNA of *E. coli* using two-step PCR and inserted into pQE80L using Exnase II. Primers for the construction of preMBP are shown in Additional file 1: Table S4. The resultant plasmid was transformed into *E. coli* JM109.

SecB, SecB_T10A_ and preMBP proteins were purified to homogeneity using nickel affinity chromatography equipped with HisTrap™ FF column (GE Healthcare Ltd, Shanghai). All three proteins could be washed with 300-mM imidazole. Furthermore, preMBP was desalted, concentrated and dissolved in buffer containing 0.5-mM guanidine hydrochloride, 10-mM HEPES and potassium acetate (pH 7.6, 150 mM) to avoid spontaneous folding.

### Isothermal titration calorimetry analysis

Interaction between SecB or SecB_T10A_ and preMBP was studied by isothermal titration calorimetry (ITC) using MicroCal PEAQ-ITC (Malvern Ltd, England). Buffer of SecB, SecB_T10A_ and preMBP proteins was exchanged with KPB buffer (pH 7.4, 100 mM) via a Superdex 75 column. Different molar ratios of SecB and preMBP, SecB_T10A_ and preMBP, were optimized and 120-μM SecB or SecB_T10A_ and 20-μM preMBP was obtained as the best molar ratio and protein loading concentration. Titration experiments were performed at 25 °C with initial injection of 0.4-μL SecB proteins and followed by 18 injections of 2.0-μL SecB proteins (36.4 μL in total) at 120-s interval. Background experiment was performed by titration of SecB into KPB buffer solution. All the experiments were carried out in triplicate.

## Additional file

**Additional file 1: Table S1.** Function of the molecular chaperones and primers used in this study for the overexpression of chaperones. **Table S2.** Primers for quantitative PCR and coexpression of SecB and SecA. **Table S3.** Primers for the saturation mutagenesis on T10 site. **Table S4.** Primers for the construction of preMBP. **Table S5.** Sequencing result of the 48 mutants in the random mutagenesis library. **Fig. S1.** SDS-PAGE analysis of the overexpression of chaperones in *Escherichia coli* JM109. **Fig. S2.** Growth curves of *Escherichia coli* JM109 strains engineered with overexpression of different chaperones. **Fig. S3.** Growth profiles of *E. coli* JM109/pQE80L, *E. coli* JM109/pQE80L-*ycdY* and *E. coli* JM109/pQE80L-*clpB* under different butanol concentrations. **Fig. S4.** (A) Fold changes of expression level of secB and secA under butanol stress. (B) Growth curves of *E. coli* JM109 co-overexpressed with SecB and SecA in the presence of different butanol concentrations. **Fig. S5.** Fold changes of expression level of SecB and SecB_T10A_. Samples were grown in 1% (v/v) butanol and induced with 0.2 mM IPTG (red column). **Fig. S6.** Growth curves of recombinant *E. coli* JM109 harboring saturation mutagenesis variants at T10 of SecB. **Fig. S7.** Maximum butanol tolerance evaluation of *E. coli* harboring SecB and SecB_T10A_. **Fig. S8.** Growth curves of *E. coli* JM109 harboring SecB and SecB_T10A_ under diverse organic solvents with different log*P* values. **Fig. S9.** SDS-PAGE analysis of the purification of SecB, SecB_T10A_ and preMBP. **Fig. S10.** Isothermal titration calorimetry analysis of SecB with preMBP and SecB_T10A_ with preMBP.

## Data Availability

All data generated or analyzed in the present study are included in this article and Additional file.

## Abbreviations

*μ*: specific growth rate
*T*: butanol tolerance
*A*: adhesion
