# Supplementary material for: Enhancing butanol tolerance of Escherichia coli reveals hydrophobic interaction of multi-tasking chaperone SecB
Source: Biotechnol Biofuels. 2019 Jun 28;12:164. doi: 10.1186/s13068-019-1507-7 (PMC6598250; doi:10.1186/s13068-019-1507-7)
Supplement: Supplementary file 1 — Additional file 1: Table S1. Function of the molecular chaperones and primers used in this study for the overexpression of chaperones. Table S2. Primers for quantitative PCR and coexpression of SecB and SecA. Table S3. Primers for the saturation mutagenesis on T10 site. Table S4. Primers for the construction of preMBP. Table S5. Sequencing result of the 48 mutants in the random mutagenesis library. Fig. S1. SDS-PAGE analysis of the overexpression of chaperones in Escherichia coli JM109. Fig. S2. Growth curves of Escherichia coli JM109 strains engineered with overexpression of different chaperones. Fig. S3. Growth profiles of E. coli JM109/pQE80L, E. coli JM109/pQE80L-ycdY and E. coli JM109/pQE80L-clpB under different butanol concentrations. Fig. S4. (A) Fold changes of expression level of secB and secA under butanol stress. (B) Growth curves of E. coli JM109 co-overexpressed with SecB and SecA in the presence of different butanol concentrations. Fig. S5. Fold changes of expression level of SecB and SecBT10A. Samples were grown in 1% (v/v) butanol and induced with 0.2 mM IPTG (red column). Fig. S6. Growth curves of recombinant E. coli JM109 harboring saturation mutagenesis variants at T10 of SecB. Fig. S7. Maximum butanol tolerance evaluation of E. coli harboring SecB and SecBT10A. Fig. S8. Growth curves of E. coli JM109 harboring SecB and SecBT10A under diverse organic solvents with different logP values. Fig. S9. SDS-PAGE analysis of the purification of SecB, SecBT10A and preMBP. Fig. S10. Isothermal titration calorimetry analysis of SecB with preMBP and SecBT10A with preMBP. [file 13068_2019_1507_MOESM1_ESM.doc]

***Submitted to: Biotechnology for Biofuels***

***Section: Bacterial genetics, physiology and metabolic engineering***

**Enhancing butanol tolerance of *Escherichia coli* reveals hydrophobic interaction of multi-tasking chaperone SecB**

Guochao Xu, Anning Wu, Lin Xiao, Ruizhi Han, Ye Ni*

*The Key Laboratory of Industrial Biotechnology, Ministry of Education, School of Biotechnology, Jiangnan University, Wuxi 214122, Jiangsu, China*

**Corresponding author, E-mail:* [*yni@jiangnan.edu.cn*](mailto:yni@jiangnan.edu.cn)

[**Table S1** Function of the molecular chaperones and primers used in this study for the overexpression of chaperones. S2](#_Toc485810890)

[**Table S2** Primers for quantitative PCR and coexpression of SecB and SecA. S4](#_Toc485810891)

[**Table S3** Primers for the saturation mutagenesis on T10 site. S5](#_Toc485810892)

[**Table S4** Primers for the construction of preMBP. S6](#_Toc485810893)

[**Table S5** Sequencing result of the 48 mutants in the random mutagenesis library. S7](#_Toc485810894)

[**Figure S1** SDS-PAGE analysis of the overexpression of chaperones in *Escherichia coli* JM109. S8](#_Toc485810895)

[**Figure S2** Growth curves of *Escherichia coli* JM109 strains engineered with overexpression of different chaperones. S9](#_Toc485810896)

[**Figure S3** Growth profiles of *E. coli* JM109/pQE80L, *E. coli* JM109/pQE80L-*ycdY* and *E. coli* JM109/pQE80L-*clpB* under different butanol concentrations. S10](#_Toc485810897)

[**Figure S4** (A) Fold changes of expression level of *secB* and *secA* under butanol stress. (B) Growth curves of *E. coli* JM109 co-overexpressed with SecB and SecA in the presence of different butanol concentrations. S11](#_Toc485810898)

[**Figure S5** Fold changes of expression level of SecB and SecB_T10A_. Samples were grown in 1% (v/v) butanol and induced with 0.2 mM IPTG (red column). S12](#_Toc485810899)

[**Figure S6** Growth curves of recombinant *E. coli* JM109 harboring saturation mutagenesis variants at T10 of SecB. S13](#_Toc485810900)

[**Figure S7** Maximum butanol tolerance evaluation of *E. coli* harboring SecB and SecB_T10A_. S14](#_Toc485810901)

[**Figure S8** Growth curves of *E. coli* JM109 harboring SecB and SecB_T10A_ under diverse organic solvents with different log*P* values. S15](#_Toc485810902)

[**Figure S9** SDS-PAGE analysis of the purification of SecB, SecB_T10A_ and preMBP. S16](#_Toc485810903)

[**Figure S10** Isothermal titration calorimetry analysis of SecB with preMBP and SecB_T10A_ with preMBP. S17](#_Toc485810904)

[**Reference** S18](#_Toc485810905)

**Table S1** Function of the molecular chaperones and primers used in this study for the overexpression of chaperones.

| **Gene & Accession No.** | **Function or putative function** | **Length**  **[bp]** | **Primer sequence (5’→3’)** |
| --- | --- | --- | --- |
| *nlpE*  P40710 | lipoprotein involved with copper homeostasis and adhesion | 711 | F: caccatcaccatcacggatccATGGTGAAAAAAGCGATAGTGACA |
|  |  |  | R: caagctcagctaattaagcttTTACTGCCCCAAACTACTGCAA |
| *clpB*  P63284 | protein disaggregation chaperone | 2130 | F: caccatcaccatcacggatccATGCGTCTGGATCGTCTTACTAATAA |
|  |  |  | R: caagctcagctaattaagcttTTACTGGACGGCGACAATCC |
| *htpG*  P0A6Z3 | molecular chaperone HSP90 family | 1875 | F: caccatcaccatcacggatccATGAAAGGACAAGAAACTCGTGG |
|  |  |  | R: caagctcagctaattaagcttTCAGGAAACCAGCAGCTGGT |
| *groL*  P0A6F5 | Cpn60 chaperonin GroEL, large subunit of GroESL | 1647 | F: caccatcaccatcacggatccATGGCAGCTAAAGACGTAAAATTCG |
|  |  |  | R: caagctcagctaattaagcttTTACATCATGCCGCCCATG |
| *grpE*  P09372 | heat shock protein | 594 | F: caccatcaccatcacggatccATGAGTAGTAAAGAACAGAAAACGCC |
|  |  |  | R: caagctcagctaattaagcttTTAAGCTTTTGCTTTCGCTACAGT |
| *lolA*  P61316 | chaperone for lipoproteins | 612 | F: caccatcaccatcacggatccATGAAAAAAATTGCCATCACCTG |
|  |  |  | R: caagctcagctaattaagcttCTACTTACGTTGATCATCTACCGTGA |
| *djlA*  P31680 | DnaJ-like protein, membrane anchored | 816 | F: caccatcaccatcacggatccATGCAGTATTGGGGAAAAATCATT |
|  |  |  | R: caagctcagctaattaagcttTCATTTAAACCCTTTCTGCTGCT |
| *bepA*  P66948 | Functions as both a chaperone and a metalloprotease | 1465 | F: caccatcaccatcacggatccATGTTCAGGCAGTTGAAAAAAAACC |
|  |  |  | R: caagctcagctaattaagcttTTACATCTTGGTATAAGGCTTAAAGCG |
| *yajL*  Q46948 | Protein deglycase 3 | 591 | F: caccatcaccatcacggatccATGAGCGCATCGGCACTG |
|  |  |  | R: caagctcagctaattaagcttCTACTCGTAATAATTATAAATCCCTGCC |
| *dnaK*  P0A6Y8 | chaperone Hsp70, co-chaperone with DnaJ | 1917 | F: caccatcaccatcacggatccATGGGTAAAATAATTGGTATCGACC |
|  |  |  | R: caagctcagctaattaagcttTTATTTTTTGTCTTTGACTTCTTCAAATT |
| *dnaJ*  P08622 | chaperone Hsp40, co-chaperone with DnaK | 1131 | F: caccatcaccatcacggatccATGGCTAAGCAAGATTATTACGAGAT |
|  |  |  | R: caagctcagctaattaagcttTTAGCGGGTCAGGTCGTCAA |
| *clpA*  P0ABH9 | ATPase and specificity subunit of ClpA-ClpP ATP-dependent serine protease, chaperone activity | 2277 | F: caccatcaccatcacggatccATGCTCAATCAAGAACTGGAACTCA |
|  |  |  | R: caagctcagctaattaagcttTTAATGCGCTGCTTCCGC |
| *cbpA*  P36659 | curved DNA-binding protein, DnaJ homologue that functions as a co-chaperone of DnaK | 921 | F: caccatcaccatcacggatccATGGAATTAAAGGATTATTACGCCA |
|  |  |  | R: caagctcagctaattaagcttTTATGCTTTCCCCCAATCTTTACG |
| *clpX*  P0A6H1 | ATPase and specificity subunit of ClpX-ClpP ATP-dependent serine protease | 1275 | F: caccatcaccatcacggatccATGACAGATAAACGCAAAGATGGC |
|  |  |  | R: caagctcagctaattaagcttTTATTCACCAGATGCCTGTTGC |
| *hscC*  P77319 | Hsp70 family chaperone Hsc62 | 1671 | F: caccatcaccatcacggatccATGGATAATGCAGAACTCGCC |
|  |  |  | R: caagctcagctaattaagcttTTATGGGATCTCAATGGCTAAAT |
| *hslO*  P0A6Y5 | heat shock protein Hsp33 | 879 | F: caccatcaccatcacggatccATGCCGCAACATGACCAATT |
|  |  |  | R: caagctcagctaattaagcttTTAATGAACTTGCGGATCTGCC |
| *ibpA*  P0C054 | heat shock chaperone | 414 | F: caccatcaccatcacggatccATGCGTAACTTTGATTTATCCC |
|  |  |  | R: caagctcagctaattaagcttTTAGTTGATTTCGATACGGCGC |
| *ibpB*  P0C058 | heat shock chaperone | 429 | F: caccatcaccatcacggatccATGCGTAACTTCGATTTATCCC |
|  |  |  | R: caagctcagctaattaagcttTTAGCTATTTAACGCGGGACG |
| *nfuA*  P63020 | iron-sulfur cluster biogenesis under severe conditions | 576 | F: caccatcaccatcacggatccATGATCCGTATTTCCGATGCTG |
|  |  |  | R: caagctcagctaattaagcttTTAGTAGTAGGAGTGTTCGCCG |
| *hchA*  P31658 | Hsp31 molecular chaperone | 852 | F: caccatcaccatcacggatccATGACTGTTCAAACAAGTAAA |
|  |  |  | R: caagctcagctaattaagcttTTAACCCGCGTAAGCTGCC |
| *ppiD*  P0ADY1 | peptidyl-prolyl cis-trans isomerase (rotamase D) | 1872 | F: caccatcaccatcacggatccATGATGGACAGCTTACGCACG |
|  |  |  | R: caagctcagctaattaagcttTTATTGCTGTTCCAGCGCATC |
| *skp*  P0AEU7 | periplasmic chaperone | 486 | F: caccatcaccatcacggatccGTGAAAAAGTGGTTATTAGCT |
|  |  |  | R: caagctcagctaattaagcttTTATTTAACCTGTTTCAGTACGT |
| *secB*  P0AG86 | protein export chaperone | 468 | F: caccatcaccatcacggatccATGTCAGAACAAAACAACACT |
|  |  |  | R: caagctcagctaattaagcttTCAGGCATCCTGATGTTCTTCA |
| *surA*  P0ABZ6 | peptidyl-prolyl cis-trans isomerase (PPIase) | 1287 | F: caccatcaccatcacggatccATGAAGAACTGGAAAACGCTG |
|  |  |  | R: caagctcagctaattaagcttTTAGTTGCTCAGGATTTTAACG |
| *ycdY*  P75915 | conserved protein | 555 | F: caccatcaccatcacggatccATGAACGAGTTTTCTATCCTCT |
|  |  |  | R: caagctcagctaattaagcttTTATTCTTCAGAATCTTCTTCCA |
| *yegD*  P36928 | putative heat shock protein | 1353 | F: caccatcaccatcacggatccGTGTTTATTGGTTTTGATTACG |
|  |  |  | R: caagctcagctaattaagcttTTAACGAAACACCACTTCCGC |
| *yrhB*  P46857 | hypothetical protein yrhB | 285 | F: caccatcaccatcacggatccATGATTACTTATCACGACGCAT |
|  |  |  | R: caagctcagctaattaagcttTCAGGGCAAGCCGAAGGT |

**Table S2** Primers for quantitative PCR and coexpression of SecB and SecA.

| **Primer** | **Sequence (5’→3’)** |
| --- | --- |
| Coexpression-*secA*-F | caccatcaccatcacggatccATGCTAATCAAATTGTTAACTAAAGT |
| Coexpression-*secA*-R | caagctcagctaattaagcttTTATTGCAGGCGGCCATGGCAC |
| qPCR-*secB*-F | AAGATTGGCAACCAGAAGTT |
| qPCR-*secB*-R | AGTGCATCACCAGCATGGTA |
| qPCR-*secA*-F | AGCCCTGAAGAACGTGTACA |
| qPCR-*secA*-R | CACCAGACCACGTTCGGTC |

**Table S3** Primers for the saturation mutagenesis on T10 site.

| **Primer** | **Sequence (5’→3’)** | **Primer** | **Sequence (5’→3’)** |
| --- | --- | --- | --- |
| T10C-F | AACACTGAAATGTGTTTCCAG | T10M-F | AACACTGAAATGATGTTCCAG |
| T10C-R | TTGGATCTGGAAACACATTTC | T10M-R | TTGGATCTGGAACATCATTTC |
| T10D-F | AACACTGAAATGGATTTCCAG | T10N-F | AACACTGAAATGAATTTCCAG |
| T10D-R | TTGGATCTGGAAATCCATTTC | T10N-R | TTGGATCTGGAAATTCATTTC |
| T10E-F | AACACTGAAATGGAATTCCAG | T10P-F | AACACTGAAATGCCGTTCCAG |
| T10E-R | TTGGATCTGGAATTCCATTTC | T10P-R | TTGGATCTGGAACGGCATTTC |
| T10F-F | AACACTGAAATGTTTTTCCAG | T10Q-F | AACACTGAAATGCAGTTCCAG |
| T10F-R | TTGGATCTGGAAAAACATTTC | T10Q-R | TTGGATCTGGAACTGCATTTC |
| T10G-F | AACACTGAAATGGGTTTCCAG | T10R-F | AACACTGAAATGCGTTTCCAG |
| T10G-R | TTGGATCTGGAAACCCATTTC | T10R-R | TTGGATCTGGAAACGCATTTC |
| T10H-F | AACACTGAAATGCATTTCCAG | T10S-F | AACACTGAAATGAGCTTCCAG |
| T10H-R | TTGGATCTGGAAATGCATTTC | T10S-R | TTGGATCTGGAAGCTCATTTC |
| T10I-F | AACACTGAAATGATTTTCCAG | T10V-F | AACACTGAAATGGTTTTCCAG |
| T10I-R | TTGGATCTGGAAAATCATTTC | T10V-R | TTGGATCTGGAAAACCATTTC |
| T10K-F | AACACTGAAATGAAATTCCAG | T10W-F | AACACTGAAATGTGGTTCCAG |
| T10K-R | TTGGATCTGGAATTTCATTTC | T10W-R | TTGGATCTGGAACCACATTTC |
| T10L-F | AACACTGAAATGCTGTTCCAG | T10Y-F | AACACTGAAATGTATTTCCAG |
| T10L-R | TTGGATCTGGAACAGCATTTC | T10Y-R | TTGGATCTGGAAATACATTTC |

**Table S4** Primers for the construction of preMBP.

| **Primer** | **Sequence (5’→3’)** |
| --- | --- |
| Pre-F | caccatcaccatcacggatccATGAGATTTCCTTCAATTTTTACTGCAGTTTTATTCGCAGCATCC |
| Pre-malE-F | CTGCAGTTTTATTCGCAGCATCCTCCGCATTAGCTATGAAAATAAAAACAGGTGCACGC |
| malE-R | caagctcagctaattaagcttTTACTTGGTGATACGAGTCTGCGC |

**Table S5** Sequencing result of the 48 mutants in the random mutagenesis library.

| Mutant | Amino acid substitution | Mutant | Amino acid substitution |
| --- | --- | --- | --- |
| 1-G12 | T115G | 10-B11 | E8V, M138R |
| 2-D12 | F107S | 11-F4 | M117I, F137S |
| 2-F4 | T10A, A73T | 11-B2 | T10A, E71G |
| 2-B2 | T10A, Q50H | 12-E4 | T10A, V59I |
| 2-E8 | E8D | 14-E10 | A73T, N139D |
| 2-H11 | T10A, V59I | 14-B1 | L136P |
| 3-A3 | T10A, F11L | 17-E8 | K34R, T115A |
| 3-A1 | T10A, D35V | 17H11 | K41E, N103H |
| 3-B5 | N139S | 18-A1 | L126P |
| 4-G11 | E3K, Q37R | 19-B1 | T115A |
| 4-G9 | T10A, C96R | 19-B10 | D45G |
| 5-F12 | T10A, I86V | 19-H8 | T10A, L141S, T149A |
| 6-B11 | M117I, F137S | 19-H9 | M94V |
| 6-G11 | F133Y | 20-E5 | T10A, V31A |
| 6-E10 | T122A | 21-H5 | A73V, E79A |
| 6-A2 | I21N | 21-F5 | F106L |
| 6-H8 | E147G | 22-G4 | T10A, V31I, N127D |
| 7-A6 | S67P, E71G | 23-C12 | S67P |
| 7-B9 | T10A, P29S | 24-H9 | T10A, Y56H |
| **7-C4** | **T10A** | 24-A4 | Y56C |
| 7-E11 | T10A/K41R | 24-A1 | T128G |
| 8-E5 | K34R | 25-F3 | A135G |
| 8-B3 | Q37L, E71G | 26-E3 | E8D |
| 9-E12 | T10A, T63A, Q125R | 26-B5 | I83T |

**Figure S1** SDS-PAGE analysis of the overexpression of chaperones in *Escherichia coli* JM109.

S: supernatant, P: precipitant.

**Figure S2** Growth curves of *Escherichia coli* JM109 strains engineered with overexpression of different chaperones.

**Figure S3** Growth profiles of *E. coli* JM109/pQE80L, *E. coli* JM109/pQE80L-*ycdY* and *E. coli* JM109/pQE80L-*clpB* under different butanol concentrations.

(●): 0.8% butanol, (■): 1.0% butanol, (◆): 1.2% butanol.

**Figure S4** (A) Fold changes of expression level of *secB* and *secA* under butanol stress. (B) Growth curves of *E. coli* JM109 co-overexpressed with SecB and SecA in the presence of different butanol concentrations. The butanol tolerance was evaluated from the OD_600_ value after 10 h of butanol treatment. All experiments were performed in triplicate.

**Figure S5** Fold changes of expression level of SecB and SecB_T10A_. Samples were grown in 1% (v/v) butanol and induced with 0.2 mM IPTG (red column). Samples grown in 1% n-butanol without induction were regarded as control (green column). All experiments were performed in triplicate.

**Figure S6** Growth curves of recombinant *E. coli* JM109 harboring saturation mutagenesis variants at T10 of SecB.

**Figure S7** Maximum butanol tolerance evaluation of *E. coli* harboring SecB and SecB_T10A_.

(A): no butanol, (B) 0.8% butanol, (C): 1.0% butanol, (D): 1.2% butanol, (E): 1.4% butanol, (F): 1.6% butanol, (G): 1.8% butanol, (H): 2.0% butanol.

(-●-): *E. coli* JM109/pQE80L, (-●-): *E. coli* JM109/pQE80L-*secB*, (-●-): *E. coli* JM109/pQE80L-*secB_T10A_.* All experiments were carried out in triplicate.

**Figure S8** Growth curves of *E. coli* JM109 harboring SecB and SecB_T10A_ under diverse organic solvents with different log*P* values.

(-●-): *E. coli* JM109/pQE80L, (-●-): *E. coli* JM109/pQE80L-*secB*, (-●-): *E. coli* JM109/pQE80L-*secB_T10A_*.


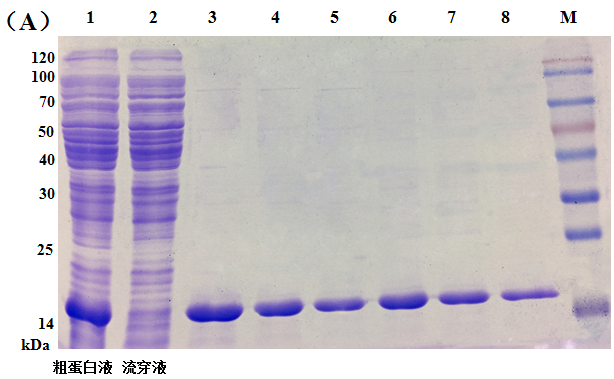


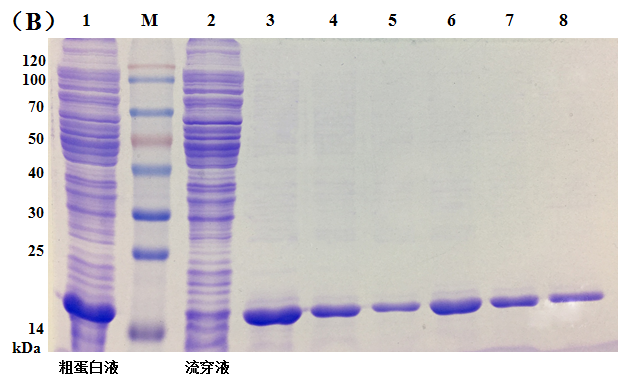


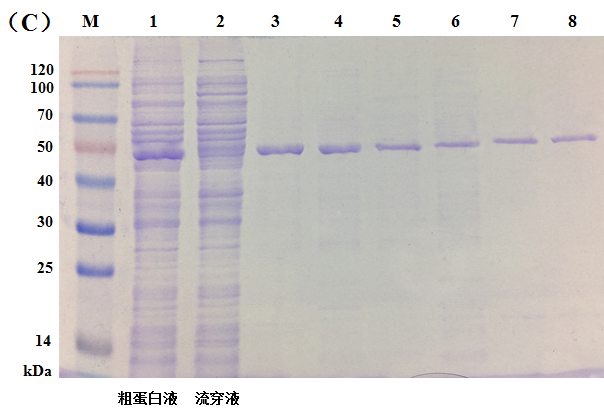


**Figure S9** SDS-PAGE analysis of the purification of SecB, SecB_T10A_ and preMBP.

M: protein molecular marker, Lane 1: supernatant; Lane 2: wash through part, Lanes 3–5: eluents of corresponding proteins under 300 mM imidazole, Lanes 6–7: eluents of corresponding proteins under 500 mM imidazole.

**Figure S10** Isothermal titration calorimetry analysis of SecB with preMBP and SecB_T10A_ with preMBP.

(A) titration curves of SecB with preMBP, (B) titration curves of SecB_T10A_ with preMBP, (C) ΔG, ΔH and –TΔS of SecB with preMBP, (D) ΔG, ΔH and –TΔS of SecB_T10A_ with preMBP.

**Reference**

1. Si, H.M., Zhang, F., Wu, A.N., Han, R.Z., Xu, G.C., Ni, Y., 2016. DNA microarray of global transcription factor mutant reveals membrane-related proteins involved in n-butanol tolerance in *Escherichia coli*. Biotechnol. Biofuels. 9, 114.
2. Bechtluft, P., Kedrov, A., Slotboom, D.J., Nouwen, N., Tans, S.J., Driessen, A.J.M., 2010. Tight hydrophobic contacts with the SecB chaperone prevent folding of substrate proteins. Biochemistry. 49, 2380–2388.
3. Knoblauch, N.T.M., Rudiger, S., Schonfeld, H.J., Driessen, A.J.M., Schneider-Mergener, J., Bukau, B., 1999. Substrate specificity of the SecB chaperone. J. Biol. Chem., 274, 34219–34225.
